# Patient-Centric Scheduling With the Implementation of Health Information Technology to Improve the Patient Experience and Access to Care: Retrospective Case-Control Analysis

**DOI:** 10.2196/16451

**Published:** 2020-06-10

**Authors:** Sukyung Chung, Meghan C Martinez, Dominick L Frosch, Veena G Jones, Albert S Chan

**Affiliations:** 1 Quantitative Sciences Unit School of Medicine Stanford University Palo Alto, CA United States; 2 Palo Alto Medical Foundation Research Institute Palo Alto, CA United States; 3 Palo Alto Medical Foundation Palo Alto, CA United States; 4 Sutter Health Sacramento, CA United States; 5 Center for Biomedical Information Research Stanford University Palo Alto, CA United States

**Keywords:** access to care, health information technology, appointment scheduling, patient-centered care

## Abstract

**Background:**

Cancellations and rescheduling of doctor’s appointments are common. An automated rescheduling system has the potential to facilitate the rescheduling process so that newly opened slots are promptly filled by patients who need and can take the slot. Building on an existing online patient portal, a large health care system adopted an automated rescheduling system, Fast Pass, that sends out an earlier appointment offer to patients via email or SMS text messaging and allows patients to reschedule their appointment through the online portal.

**Objective:**

We examined the uptake of Fast Pass at its early stage of implementation. We assessed program features and patient and visit characteristics associated with higher levels of Fast Pass utilization and the association between Fast Pass use and no-show and cancellation rates.

**Methods:**

This study was a retrospective analysis of Fast Pass offers sent between July and December 2018. Multivariable logistic regression was used to assess the independent contribution of program, patient, and visit characteristics on the likelihood of accepting an offer. We then assessed the appointment outcome (completion, cancellation, or no-show) of Fast Pass offered appointments compared to appointments with the same patient and visit characteristics, but without an offer.

**Results:**

Of 177,311 Fast Pass offers sent, 14,717 (8.3%) were accepted. Overall, there was a 1.3 percentage point (38%) reduction in no-show rates among Fast Pass accepted appointments compared to other appointments with matching characteristics (*P*<.001). The offers were more likely to be accepted if they were sent in the evening (versus early morning), the first (versus repeated) offer for the same appointment, for a slot 1-31 days ahead (versus same-day), for later in a day (versus before 10am), for a primary care (versus specialty) visit, sent via SMS text messaging (versus email only), for an appointment made through the online patient portal (versus via phone call or in-person), or for younger adults aged 18-49 years (versus those aged 65 years or older; all at *P*<.001). Factors negatively associated with offer acceptance were a higher number of comorbidities (*P*=.02) and visits scheduled for chronic conditions (versus acute conditions only; *P*=.002).

**Conclusions:**

An automated rescheduling system can improve patients’ access by reducing wait times for an appointment, with an added benefit of reducing no-shows by serving as a reminder of an upcoming appointment. Future modifications, such as increasing the adoption of SMS text messaging offers and targeting older adults or patients with complex conditions, may make the system more patient-centered and help promote wider utilization.

## Introduction

Physicians and health care systems have long struggled with two concomitant but discordant conditions with regard to patient access to care. Many health systems simultaneously report long wait times for patient appointments and available appointment slots that are underutilized. Common contributing factors to this are last-minute cancellations and appointment no-shows by patients, leaving unfilled appointment slots in a physician’s schedule. No-show rates have been variably reported to be as low as 2% to more than 50% of scheduled appointments [1-6], and represent an estimated annual cost to the health care system of $150 billion [6-8]. These schedule holes disrupt clinic workflows and reduce efficiency, reduce access for other patients who could have filled the slot, represent lost revenue for the health care systems, result in worse patient health outcomes, and ultimately cause dissatisfaction for patients and health care providers [9-13]. Typical countermeasures are often clinician-centric. For example, clinicians may maintain long wait queues to increase the probability of full schedules. Clinicians have relied on staff to manage waitlists of patients, but this approach is time- and labor-intensive, as clinic staff spend time calling patients to try to fill open slots [1,14,15].

Health information technology (HIT), particularly electronic health records (EHR), is often cited as a source of physician burnout, with clinical documentation requirements and other administrative tasks associated with EHR use in the United States being major contributing factors [16-18]. However, this literature often fails to recognize the counterbalancing beneficial aspects of HIT, such as enabling patients to access their health records electronically, schedule and reschedule appointments, and actively participate in shared decision-making, potentially resulting in improved care experiences and satisfaction [19-22]. An automated HIT system that leverages digital patient engagement to take advantage of available care options presents an opportunity to create a mutually beneficial scenario, addressing the need to improve practice efficiency and the patients’ desire for more timely access.

In 2015, Sutter Health began piloting an automated appointment offer and fulfillment program called Fast Pass, a module developed by Epic Systems Corporation, and subsequently completed an enterprise-wide implementation in ambulatory primary care and specialty practices in 2018. Fast Pass allows a waitlisted patient to receive an automated message when an earlier appointment slot is available, and accept it or keep the original appointment. Fast Pass has significant potential to improve access and patient experience, in addition to promoting efficiency by filling clinic slots.

We therefore conducted a rigorous evaluation of the Fast Pass system. We evaluated the implementation of the system in 2018 after it had been rolled out across the Sutter Health system. The current paper describes the program features and identifies patient and visit factors associated with higher levels of Fast Pass utilization, as well as the association between Fast Pass implementation and no-show and cancellation rates. To our knowledge, this is the first study to systematically evaluate the implementation and effectiveness of an automated rescheduling system across ambulatory primary and specialty care settings.

## Methods

### Setting

Sutter Health is a large, not-for-profit health care system in Northern California serving more than 3 million people across 100 rural, suburban, and urban communities. The health care population represents high diversity in insurance coverage and race/ethnicity (eg, 17% Asian, 11% Hispanic/Latino), socio-demographics, and cultural backgrounds, mirroring the larger underlying catchment area. The health care system has a long history of utilizing HIT, with one affiliate becoming the first health system in the nation to implement Epic Systems Corporation’s MyChart patient portal, My Health Online (MHO), in 2001.

### Features of Fast Pass

In late 2015, Sutter implemented a pilot program, Fast Pass, to allow patients to be notified via email of an earlier appointment slot should one become available. Then, in early 2018, along with implementation of SMS text messaging reminders for appointments, Fast Pass was rolled out across the organization and patients were allowed to opt in to receiving Fast Pass notices via email, SMS text messaging, or both.

**Figure 1:**
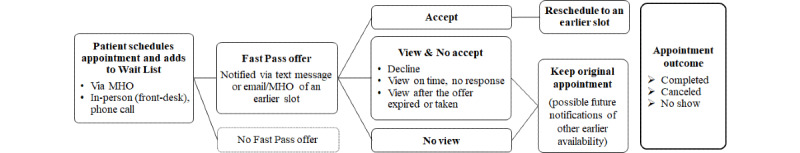
Flow of scheduling, rescheduling, and appointment outcomes with Fast Pass. MHO: My Health Online.

To use Fast Pass, a patient must first opt into the program via MHO (the online patient portal) and elect to receive notifications via email, SMS text messaging, or both (Figure 1). After a patient schedules an appointment, they can elect to be added to the waitlist for an earlier appointment slot. Once notified of an earlier slot, the patient must log on to MHO to respond. After several modifications during the initial pilot phase, the current Fast Pass system is scheduled to offer alternative appointment slots 9 times per day (Table 1). Two of these batches are sent early in the morning between 6 AM and 7:30 AM for same-day appointments and they expire after 30 minutes. One batch is sent midday (11:30 AM), also expires after 30 minutes, and is for appointment slots either that same day or up to 7 days ahead. The final six batches are all sent in the evening between 6 PM and 8:30 PM for appointments the next day up to 31 days ahead, and these offers all expire at 5:30 AM the following day. For each batch, an appointment slot was offered to 5 patients simultaneously; the offer expired automatically once it was taken on a first come, first served basis or when the time expired.

**Table 1:** Fast Pass offer characteristics.

	Batch 1	Batch 2	Batch 3	Batch 4	Batch 5	Batch 6	Batch 7	Batch 8	Batch 9
Time sent	6 AM	6:30 AM	11:30 AM	6 PM	6:30 PM	7 PM	7:30 PM	8 PM	8:30 PM
Time before expiration	30 minutes	30 minutes	30 minutes	11.5 hours	11 hours	10.5 hours	10 hours	9.5 hours	9 hours
Offered appointment date	Same day	Same day	Same day to 7 days ahead	1-31 days ahead	1-31 days ahead	1-31 days ahead	1-31 days ahead	1-31 days ahead	1-31 days ahead
Minimum days saved	1	1	1	3	3	3	3	3	3
Number of offers sent per slot	5	5	5	5	5	5	5	5	5

### Statistical Analysis

#### Factors Associated With the Likelihood of Accepting a Fast Pass Offer

We used Fast Pass offers sent between July and December 2018 for this retrospective analysis. First, we examined the distribution of responses to Fast Pass offers, classified into the following: accepted, declined, viewed on time (while the offer was active) but did not respond, viewed too late (after expired or taken by another person), and did not view.

We then assessed differences in acceptance rates based on Fast Pass feature, visit type, and patient demographic and clinical characteristics. *T* test and analysis of variance (*F* test) were used to examine differences in the unadjusted proportion of accepted offers across subgroups. Multivariable logistic regression was used to assess the independent contribution of each factor on the likelihood of accepting the offer.

#### Fast Pass and the Likelihood of No-Shows

We assessed whether outcomes of an appointment (ie, completed, canceled, or no-show) with a Fast Pass offer (whether the appointment was rescheduled through Fast Pass or the original appointment was kept) differed from appointments without an offer. We considered a canceled appointment as happening any time prior to the scheduled appointment time. Recognizing that the impact of Fast Pass would depend on patients’ responses to the offer, we analyzed two types of responses to the offer separately: “accepted” and “viewed but not accepted”. For each Fast Pass offer that was accepted or viewed, we randomly selected 5 matched appointments from the pool of patients who were actively using MHO but were not offered a Fast Pass alternative for an upcoming appointment. The Fast Pass offer appointments and 5 matching no-offer appointments were exactly matched on the following characteristics: age group (0-17, 18-39, 40-54, 55-64, 65 years or older); primary care versus specialty care; scheduled appointment time (before 10 AM, 10 AM to 11:59 AM, noon to 2:59 PM, 3 PM and later); and days between appointment made and visit date (0, 1-7, 8-24, 29 days or longer). We chose the 1:5 ratio to make the best use of available data while also reducing bias, as there were very few appointments with an offer relative to those without an offer that had the same observed characteristics [23,24].

Analyses were conducted with Stata 14.2 (StataCorp). The study was reviewed and deemed to be quality improvement by the Sutter Health Institutional Review Board.

## Results

### Responses to Fast Pass Offers

Of 177,311 Fast Pass offers sent for 44,792 appointment slots to 38,361 patients, 8.3% (n=14,717) were accepted, 11.1% (n=19,682) were declined, 8.0% (n=14,185) were viewed on time but with no response, 53.3% (n=94,507) were viewed after the offer expired or became unavailable, and 19.4% (n=34,398) were never viewed.

### Characteristics of Fast Pass Offers

Out of the 177,311 Fast Pass offers sent, most were for same-day appointments (n=48,502, 27%), appointments the next day (n=28,370, 16%), or appointments 2-7 days ahead (n=62,059, 35%), with the remainder (n=37,235, 21%) for appointments 8-31 days ahead (Table 2). If they were accepted, Fast Pass offers would have saved 8-30 days from the originally scheduled appointment in most cases (n=83,891, 47%), followed by 0-7 days (n=51,469, 29%), and 31-358 days (n=42,196, 24%). Those who accepted an offer saw their clinician on average 14.8 (SD 14.6) days sooner than initially scheduled for primary care, and 23.7 (SD 23.0) days sooner for specialty care. A quarter of offers (n=40,992, 23%) were sent during the early morning, 64,005 (36%) were sent midday, and 72,313 (41%) were sent in the evening. Fast Pass offers can be sent repeatedly for the same appointment slot, and 59,238 (33%) of appointment slots had an offer 5 or more times. See Table 2 for other patient and visit characteristics.

**Table 2:** Program, visit, and patient characteristics of Fast Pass offers.

Variables			Frequency, n (%)		Acceptance rate, n (%)		*P* value	
**Overall**			177,311 (100)		14,717 (8.30)		N/A	
	**Previous offers for the appointment**							
		0	43,692 (25)		7733 (17.70)		Ref	
		1	27,706 (16)		2798 (10.10)		<.001	
		2	19,971 (11)		1498 (7.50)		<.001	
		3	15,040 (8)		857 (5.70)		<.001	
		4	11,664 (7)		537 (4.60)		<.001	
		≥5	59,238 (33)		1244 (2.10)		<.001	
	**Time offer was made**							
		6 AM to 7:20 AM	40,992 (23)		1435 (3.50)		Ref	
		11:30 AM to 11:55 AM	64,005 (36)		3840 (6.00)		<.001	
		6 PM to 8:55 PM	72,313 (41)		9473 (13.10)		<.001	
	**Days to new appointment**							
		0	48,502 (27)		1795 (3.70)		Ref	
		1	28,370 (16)		2326 (8.20)		<.001	
		2-7	62,059 (35)		5709 (9.20)		<.001	
		8-14	14,185 (8)		1915 (13.50)		<.001	
		15-21	10,639 (6)		1340 (12.60)		<.001	
		22-31	12,412 (7)		1539 (12.40)		<.001	
	**Days saved**							
		0-7	51,469 (29)		4632 (9.00)		Ref	
		8-30	83,891 (47)		7215 (8.60)		.01	
		31-358	42,196 (24)		2912 (6.90)		<.001	
	**Scheduled via My Health Online (MHO)**							
		Offline	56,816 (32)		4204 (7.40)		Ref	
		MHO	120,495 (68)		12,411 (10.30)		<.001	
	**Opted to receive SMS text messaging**							
		No SMS text messaging	122,345 (69)		9053 (7.40)		Ref	
		SMS text messaging	54,966 (31)		5717 (10.40)		<.001	
	**Time of day of offered slot**							
		6 AM to 9:59 AM	56,740 (32)		4539 (8.00)		Ref	
		10 AM to 11:59 AM	44,328 (25)		3546 (8.00)		.91	
		Noon to 2:59 PM	46,101 (26)		3965 (8.60)		<.001	
		3 PM to 7 PM	31,916 (18)		2809 (8.80)		<.001	
	**Provider specialty**							
		Primary care	69,833 (40)		5657 (8.10)		Ref	
		Specialty care	117,219 (66)		9846 (8.40)		.009	
	**Visit type**							
		E/M^a^, acute and chronic Dxs	23,050 (13)		2858 (12.40)		Ref	
		E/M, acute Dxs only	19,504 (11)		3218 (16.50)		<.001	
		E/M, chronic Dxs only	8866 (5)		1206 (13.60)		<.001	
		Preventive visit	10,639 (6)		1266 (11.90)		.23	
		Unknown^b^	115,252 (65)		6224 (5.40)		<.001	
	**Charlson Comorbidity Index**							
		0	140,076 (79)		11,906 (8.50)		Ref	
		1	21,277 (12)		1787 (8.40)		.93	
		≥2	17,731 (10)		1188 (6.70)		<.001	
	**Age (years)**							
		0-17	11,612 (7)		743 (6.40)		Ref	
		18-49	76,777 (43)		7447 (9.70)		<.001	
		50-64	45,561 (26)		3690 (8.10)		<.001	
		≥65	42,727 (24)		2735 (6.40)		.92	
	**Sex**							
		Male	57,542 (32)		4891 (8.50)		Ref	
		Female	119,135 (67)		9769 (8.20)		<.001	
	**Race/ethnicity**							
		Non-Hispanic white	100,414 (57)		8134 (8.10)		Ref	
		African American	6176 (3)		476 (7.70)		.24	
		Asian	30,863 (17)		2778 (9.00)		<.001	
		Latino/Hispanic	19,170 (11)		1591 (8.30)		.44	
		Other race or race unknown	20,041 (11)		1704 (8.50)		.08	
**Same-day or next-day slot offers**			60,206 (100)		3131 (5.2)		N/A	
	**Time offer was made**							
		6 AM to 7:20 AM	43,692 (68)		1433 (3.50)		Ref	
		11:30 AM to 11:55 AM	27,706 (13)		344 (4.40)		.001	
		6 PM to 8:55 PM	19,971 (20)		1385 (11.50)		<.001	
	**Visit type**							
		E/M, acute and chronic Dxs	9031 (15)		768 (8.50)		Ref	
		E/M, acute Dxs only	7225 (12)		881 (12.20)		<.001	
		E/M, chronic Dxs only	3010 (5)		262 (8.70)		.69	
		Preventive visit	3612 (6)		275 (7.60)		.045	
		Unknown^b^	37,328 (62)		971 (2.60)		<.001	

^a^E/M: evaluation and management coding.

^b^This category includes visits with no diagnosis falling into acute or chronic conditions and appointments later canceled or no-shows.

### Bivariate Analysis of Characteristics of Accepted Fast Pass Offers

The first offer for an appointment slot was far more likely to be accepted than subsequent offers for the same original appointment (17.7% versus 2.1%-10.1% of acceptance rates, respectively; *P*<.001; Table 2). Offers sent in the evening were more than twice as likely to be accepted (13.1%) than morning offers (3.5%-6.0%; *P*<.001). There was a higher acceptance rate when the offered slot was more than one week ahead (12.4%-13.5%), 2-7 days ahead (9.2%), or the next day (8.2%), as compared to the same day (3.7%; *P*<.001). The acceptance rate was higher for offers with potentially fewer days saved (9.0% for 0-7 days versus 6.9% for 31-358 days; *P*<.001) and when the offered slot was in the afternoon (8.6%-8.8%) rather than in the morning (8.0%; *P*<.001). Acceptance rates were higher when the appointment was scheduled through MHO versus offline (10.3% versus 7.4%; *P*<.001) and among patients who opted to be contacted through SMS text messaging versus email only (10.4% versus 7.4%; *P*<.001). Compared to non-Hispanic whites, Asians were more likely to accept an offer (9.0% versus 8.1%; *P*<.001).

Patients with a high comorbidity burden were less likely to accept offers (Charlson Comorbidity Index [CCI]=0, 8.5%; CCI≥2, 6.7%; *P*<.001). Acceptance rates were highest among people aged 18-49 years (9.7%) and lower for those aged 0-17 years (6.4%; their guardian received the messages) or 50-64 years (8.1%; *P*<.001). Male patients were more likely to accept an offer than female patients (8.5% versus 8.2%; *P*<.001). Accepted visits were more likely to be for acute conditions only (16.5%) rather than for chronic conditions only (13.6%) or both acute and chronic conditions (12.4%; *P*<.001). Specialty care visits were slightly more likely to be accepted (8.4% versus 8.1%; *P*=.009), and there were no differences in acceptance rates across specialties (eg, dermatology, ob-gyn, orthopedics).

For same day or next-day offers only (N=60,206), offers sent in the evening (of the day before the opened slot) rather than in the morning (of the appointment day) were three times more likely to be accepted (11.5% versus 3.5%; *P*<.001), and patients with acute conditions only (12.2%) were more likely to accept the offer (*P*<.001) than those with both chronic and acute conditions (8.5%).

### Factors Associated With Offer Acceptance in Multivariable Analysis

After controlling all other factors, findings from the adjusted, multivariable analysis (Table 3) were similar to those from the unadjusted, bivariate analysis, with a few exceptions. Preventive visit, older age (aged 65 years or older), and specialty visit were negative predictors of Fast Pass acceptance (*P*<.001). In addition, the following factors no longer had a significant association with offer acceptance in the adjusted model: potentially saved at least 31 days (versus 7 days or fewer; *P*=.33), CCI≥2 (*P*=.24), visit for chronic conditions (versus both acute and chronic conditions; *P*=.48), female (*P*=.04), aged 50-64 years (versus 0-17; *P*=.17), and Asian (versus non-Hispanic white; *P*=.08).

**Table 3:** Predictors of Fast Pass offer acceptance from a logistic regression (N=176,615)^a^.

	Variables	Odds ratio	99% CI	*P* value
**Previous offers for the same appointment**				
	0	Ref		
	1	0.55	0.52-0.59	<.001
	2	0.40	0.37-0.43	<.001
	3	0.30	0.27-0.33	<.001
	4	0.24	0.22-0.27	<.001
	≥5	0.11	0.10-0.12	<.001
**Time offer was made**				
	6 AM to 7:20 AM	Ref		
	11:30 AM to 11:55 AM	1.19	1.01-1.41	.006
	6 PM to 8:55 PM	2.30	1.92-2.76	<.001
**Days to new appointment**				
	0	Ref		
	1	1.58	1.34-1.86	<.001
	2-7	1.84	1.57-2.15	<.001
	8-14	2.04	1.71-2.43	<.001
	15-21	1.93	1.60-2.33	<.001
	22-31	1.91	1.59-2.30	<.001
**Days saved**				
	0-7	Ref		
	8-30	1.06	1.00-1.12	.02
	31-358	0.97	0.90-1.05	.33
**Scheduled via My Health Online (MHO)**				
	Offline	Ref		
	MHO	1.31	1.22-1.40	<.001
**Opted to receive SMS text messaging**				
	No SMS text messaging	Ref		
	SMS text messaging	1.46	1.37-1.55	<.001
**Time of day of offered slot**				
	6 AM to 9:59 AM	Ref		
	10 AM to 11:59 AM	1.12	1.05-1.20	<.001
	Noon to 2:59 PM	1.16	1.09-1.24	<.001
	3 PM to 7 PM	1.26	1.18-1.36	<.001
**Provider specialty**				
	Primary care	Ref		
	Specialty care	0.81	0.76-0.87	<.001
**Visit type**				
	E/M, acute and chronic Dxs	Ref		
	E/M, acute Dxs only	1.13	1.02-1.26	.002
	E/M, chronic Dxs only	1.04	0.90-1.20	.48
	Preventive visit	0.76	0.67-0.87	<.001
	Unknown	0.28	0.25-0.30	<.001
**Charlson Comorbidity Index**				
	0	Ref		
	1	1.08	0.99-1.18	.02
	≥2	0.95	0.85-1.06	.24
**Age (years)**				
	0-17	Ref		
	18-49	1.34	1.17-1.53	<.001
	50-64	1.08	0.94-1.24	.17
	≥65	0.81	0.69-0.93	<.001
**Sex**				
	Male	Ref		
	Female	0.95	0.90-1.01	.04
**Race/ethnicity**				
	Non-Hispanic white	Ref		
	African American	0.99	0.83-1.18	.91
	Asian	0.95	0.87-1.03	.08
	Hispanic/Latino	0.99	0.90-1.09	.73
	Other race or race unknown	0.95	0.87-1.04	.15

^a^Indicators of clinic location were also included but not presented here.

### Fast Pass and the Likelihood of Appointment Completion, Cancellation, and No-Shows

**Figure 2:**
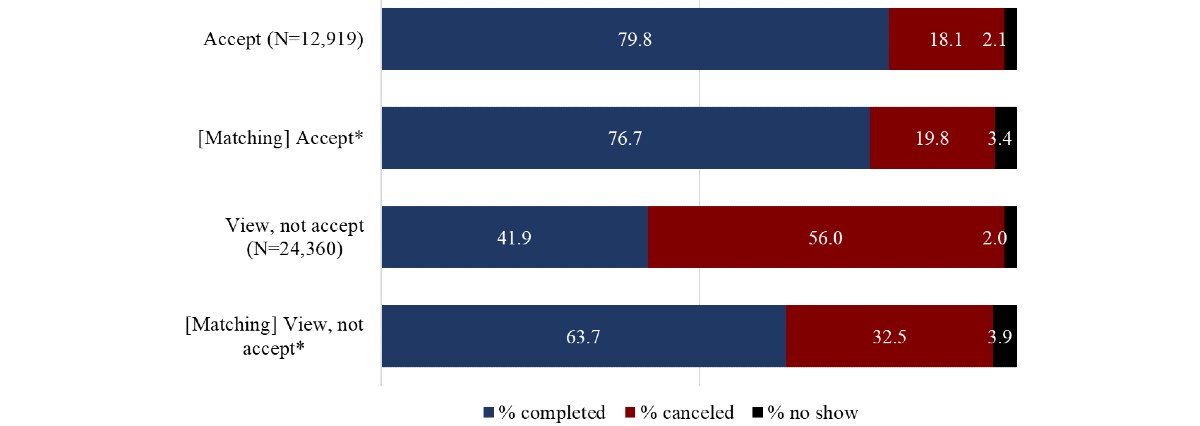
Appointment outcomes by Fast Pass offer status. * indicates matching comparison groups of "accept" and "view, not accept" groups, respectively.

As compared to appointments with matched characteristics but without a Fast Pass offer, accepted Fast Pass appointments were more likely to be completed (79.8% versus 76.7%) and less likely to be canceled (18.1% versus 19.8%) or result in no-shows (2.1% versus 3.4%; *P*<.001; Figure 2). Additionally, Fast Pass offers viewed but not accepted were less likely to be completed (41.9% versus 63.7%) and more likely to be canceled (56.0% versus 32.5%) but less likely to be no-shows (2.0% versus 3.9%; *P*<.001).

## Discussion

### Principal Findings

Fast Pass, a patient-centered application of HIT, mutually benefits patients and providers. Patients who accepted a Fast Pass offer experienced more timely access to care, with time to see a doctor reduced by 15 days in primary care and 24 days in specialty care, on average. From the provider perspective, Fast Pass offers facilitated efficient management of office visit slots by automating rescheduling processes and filling gaps in provider schedules in a timely manner.

The Fast Pass system also serves to remind the patient about an upcoming appointment, reducing no-shows by 1.3 percentage points (a 38% relative reduction or 168 appointments) and facilitating timely cancellation of appointments that are no longer needed by 1.7 percentage points (a 9% relative reduction or 219 appointments), when the offer was accepted. Even when the original appointment was kept, those who viewed the offer had almost half the no-show rate as their matched comparisons (2.0% versus 3.9%). Other studies have found that patients often miss an appointment simply because they have forgotten about it, because of long delays between scheduling an appointment and the actual visit date, or because patients have no means of cancelling [1,4,6,25,26]. Using SMS text messaging to send reminders for upcoming appointments has been shown to reduce no-show rates and increase cancellation rates, providing time for the health care system to fill that slot with another patient [1,14,15]. Though the change in no-show rates seems relatively modest, if all patients in the health care system opt into the program, it would have significant clinical implications. Applying the rate to the volume of appointments in this health care system, approximately 20 million in 2018, it translates to 260,000 no-shows that could have been avoided annually if all patients were reminded through the Fast Pass system.

Despite the potential benefits, the acceptance of Fast Pass offers was 8% of the total early appointments offered. Notably, Fast Pass offers were more likely to be accepted when the initial notification was received via SMS text messaging. The alternative to SMS text messaging of Fast Pass offers is email, which may explain the high rate of Fast Pass offers (53.3%) that were viewed after the offer expired or became unavailable. In light of protections provided by the Telephone Consumer Protection Act, which implements consumer protections from unwelcome solicitations via phone and text messaging, the organization has offered this feature to patients who have individually enrolled and consented to receiving reminders via SMS text messaging. Balancing the activation inertia of enrolling in a new program and the desire to advocate for robust consumer protections versus the desire to maximize the potential patient benefits comes with the cost of increased socio-behavioral and technical barriers to adoption. Alternative approaches not being tested here (eg, more automated enrollment for digital waitlists and SMS text messaging as well as tighter integration with conditions of registration workflows) should also be considered and evaluated in future modifications.

There are several potentially modifiable program features to improve the benefits realized from Fast Pass for those who opted into the program. Our findings suggest that offer acceptance can improve by increasing the rate of patients enrolled to receive offers delivered by SMS text messaging; sending next-day offers in the evening rather than same-day offers in the early morning; sending an offer once per appointment slot and, if not accepted, not sending another one, but instead offering it to another person; allowing more time to respond for same-day slots; and focusing on users of the online patient portal, who are more likely to view and respond to the offer. A potential modification that might help improve the acceptance rate and make the system more patient-centered is to ask patients their preference for alternative slots at the time they sign up for the waitlist. This may become feasible as more patients sign up for Fast Pass and the volume of waitlist participants is large enough to identify matching between available and preferred slots.

It is unclear, though, whether Fast Pass offers are currently utilized by those who need it most. Patients who perceive their conditions as urgent may be more likely to accept the offer, as shown with the higher acceptance rate among patients with acute conditions only and a lower rate for preventive visits. On the other hand, the acceptance rate was lower for those with multiple comorbid conditions and higher disease burdens and for older patients whose medical needs are typically greater. One potential explanation is that older adults and patients with multiple comorbidities are less likely to opt for SMS text messaging than email. To improve overall uptake of the system as well as to reduce disparities in the utilization, more outreach efforts may be necessary, especially to those who could benefit most from the system, eg, patients with higher comorbidity burdens.

### Limitations

Our study has several limitations that future extended studies should address. First, though we were able to quantitatively measure the rates of acceptances and declines of Fast Pass offers, we do not understand facilitators and barriers to using the Fast Pass program from the perspective of the patient. Focus groups or patient surveys would better examine the factors that impact adoption of the program. Second, we have yet to assess whether the system reduced the workload related to rescheduling for front desk staff and schedulers, as intended. Finally, we investigated one form of an automated rescheduling system, suggesting potential modifications for improvement. A prospective study, preferably with natural experiments, should address whether such modifications would help facilitate better utilization of the system by patients who could benefit most.

### Conclusions

Fast Pass, an innovative automated rescheduling system embedded in the EHR, improves patient access to care by reducing wait time for an appointment, with an added benefit of reducing no-shows and increasing timely appointment cancellation by serving as a reminder of an upcoming appointment. Future modifications, such as allowing more time to respond to the offer and targeting patients with complex conditions, may help enhance the value of the system and wider adoption of the model beyond the Sutter Health system. Identifying opportunities for the implementation of interventions such as Fast Pass can provide evidence of the full range of benefits of HIT by simultaneously improving clinician practice efficiency and patient experience and access to care.

## Abbreviations

CCI: Charlson Comorbidity Index
HIT: health information technology
MHO: My Health Online
